# Supplementary material for: RNA-Seq and Electrical Penetration Graph Revealed the Role of Grh1-Mediated Activation of Defense Mechanisms towards Green Rice Leafhopper (Nephotettix cincticeps Uhler) Resistance in Rice (Oryza sativa L.)
Source: Int J Mol Sci. 2021 Oct 2;22(19):10696. doi: 10.3390/ijms221910696 (PMC8509599; doi:10.3390/ijms221910696)
Supplement: Supplementary file 1 [file ijms-22-10696-s001.zip › ijms-1375403-supplementary.pdf]

Supplementary materials

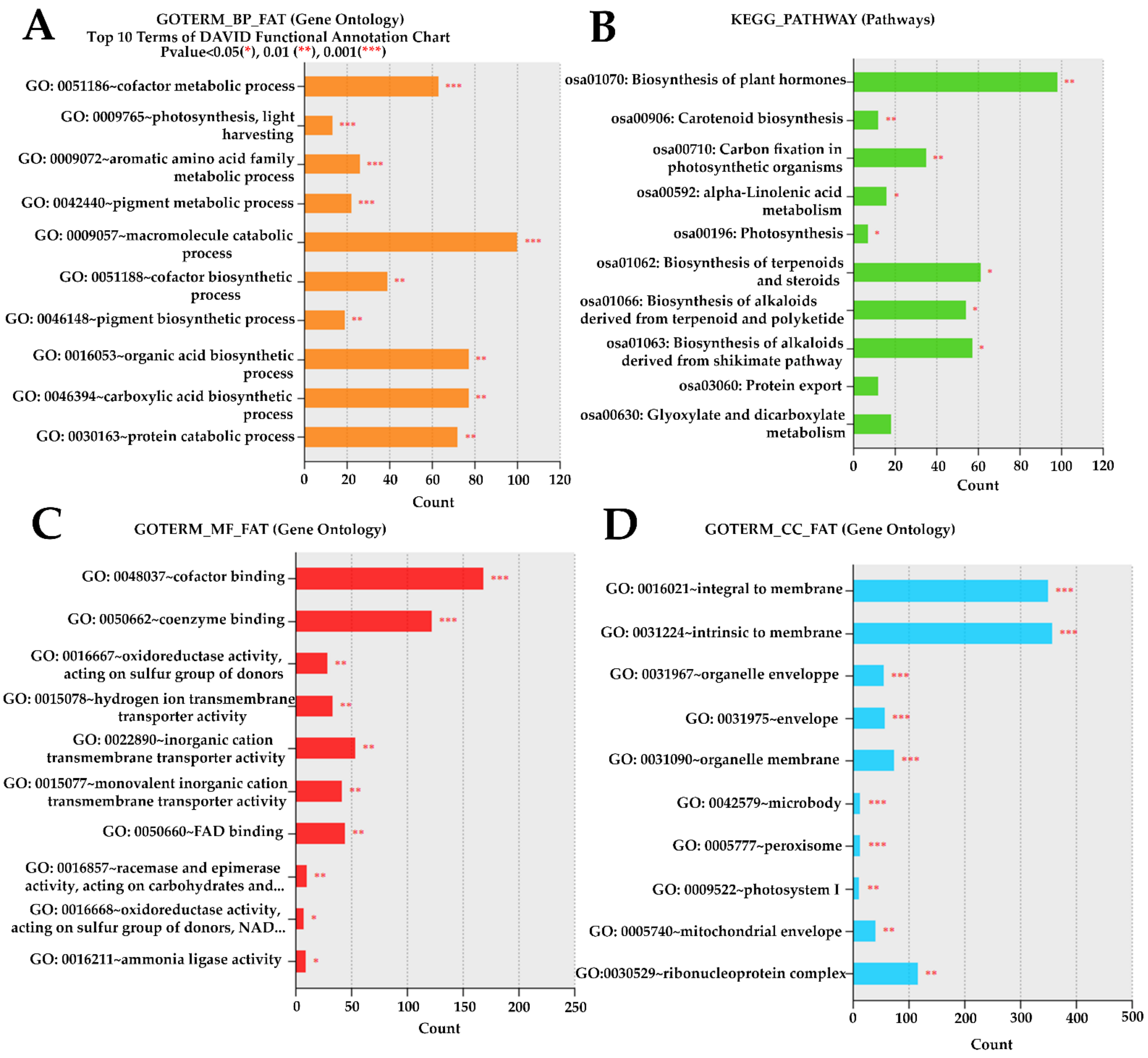

Figure S1. Gene ontology (GO) terms of DAVID annotation. Top 10 terms of DAVID functional annotation chart for (**A**) biological process (BP), (**B**) KEGG (Kyoto Encyclopedia of Genes and Genomes) pathway, (**C**) molecular function, and (**D**) cellular component. **p<0.05, **p<0.01, ***p<0.001.*

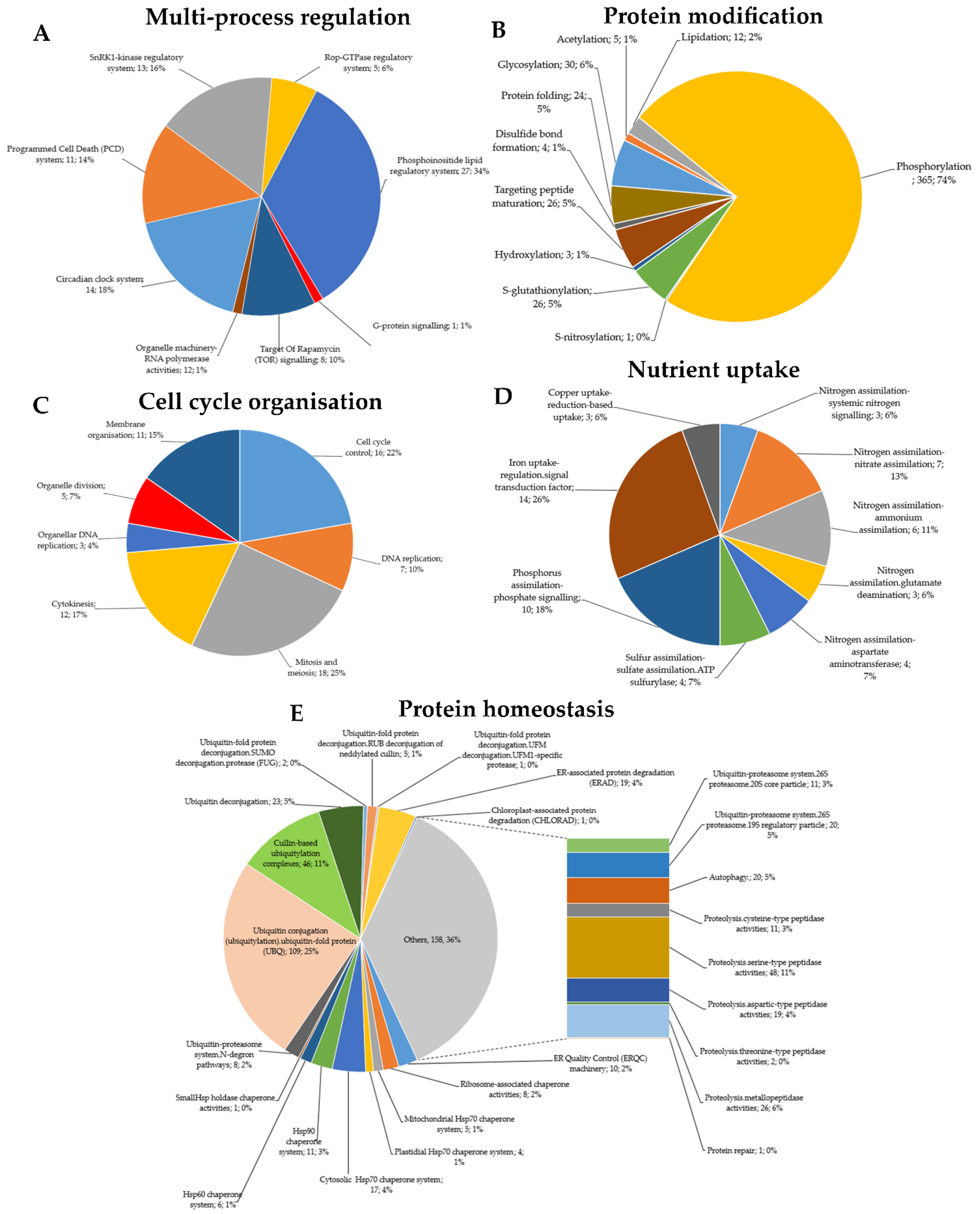
A
Multi-process regulation
SnRK1-kinase regulatory system; 13; 16%
Rop-GTPase regulatory system; 5; 6%
Programmed Cell Death (PCD) system; 11; 14%
Phosphoinositide lipid regulatory system; 27; 34%
Circadian clock system; 14; 18%
G-protein signalling; 1; 1%
Organelle machinery-RNA polymerase activities; 12; 1%
Target Of Rapamycin (TOR) signalling; 8; 10%
B
Protein modification
Acetylation; 5; 1%
Lipidation; 12; 2%
Glycosylation; 30; 6%
Protein folding; 24; 5%
Disulfide bond formation; 4; 1%
Phosphorylation ; 365; 74%
Targeting peptide maturation; 26; 5%
Hydroxylation; 3; 1%
S-glutathionylation; 26; 5%
S-nitrosylation; 1; 0%
C
Cell cycle organisation
Membrane organisation; 11; 15%
Cell cycle control; 16; 22%
Organelle division; 5; 7%
Organellar DNA replication; 3; 4%
DNA replication; 7; 10%
Cytokinesis; 12; 17%
Mitosis and meiosis; 18; 25%
D
Nutrient uptake
Copper uptake-reduction-based uptake; 3; 6%
Nitrogen assimilation-systemic nitrogen signalling; 3; 6%
Iron uptake-regulation.signal transduction factor; 14; 26%
Nitrogen assimilation-nitrate assimilation; 7; 13%
Nitrogen assimilation-ammonium assimilation; 6; 11%
Phosphorus assimilation-phosphate signalling; 10; 18%
Nitrogen assimilation.glutamate deamination; 3; 6%
Nitrogen assimilation-aspartate aminotransferase; 4; 7%
Sulfur assimilation-sulfate assimilation.ATP sulfurylase; 4; 7%
E
Protein homeostasis
Ubiquitin-fold protein deconjugation.SUMO deconjugation.protease (FUG); 2; 0%
Ubiquitin-fold protein deconjugation.RUB deconjugation of neddylated cullin; 5; 1%
Ubiquitin-fold protein deconjugation.UFM deconjugation.UFM1-specific protease; 1; 0%
ER-associated protein degradation (ERAD); 19; 4%
Ubiquitin-proteasome system.26S proteasome.20S core particle; 11; 3%
Ubiquitin deconjugation; 23; 5%
Chloroplast-associated protein degradation (CHLORAD); 1; 0%
Ubiquitin-proteasome system.26S proteasome.19S regulatory particle; 20; 5%
Cullin-based ubiquitylation complexes; 46; 11%
Autophagy.; 20; 5%
Proteolysis.cysteine-type peptidase activities; 11; 3%
Ubiquitin conjugation (ubiquitylation).ubiquitin-fold protein (UBQ); 109; 25%
Others, 158, 36%
Proteolysis.serine-type peptidase activities; 48; 11%
Proteolysis.aspartic-type peptidase activities; 19; 4%
Ubiquitin-proteasome system.N-degron pathways; 8; 2%
ER Quality Control (ERQC) machinery; 10; 2%
Proteolysis.threonine-type peptidase activities; 2; 0%
Ribosome-associated chaperone activities; 8; 2%
SmallHsp holdase chaperone activities; 1; 0%
Proteolysis.metallopeptidase activities; 26; 6%
Mitochondrial Hsp70 chaperone system; 5; 1%
Hsp90 chaperone system; 11; 3%
Plastidial Hsp70 chaperone system; 4; 1%
Protein repair; 1; 0%
Cytosolic Hsp70 chaperone system; 17; 4%
Hsp60 chaperone system; 6; 1%

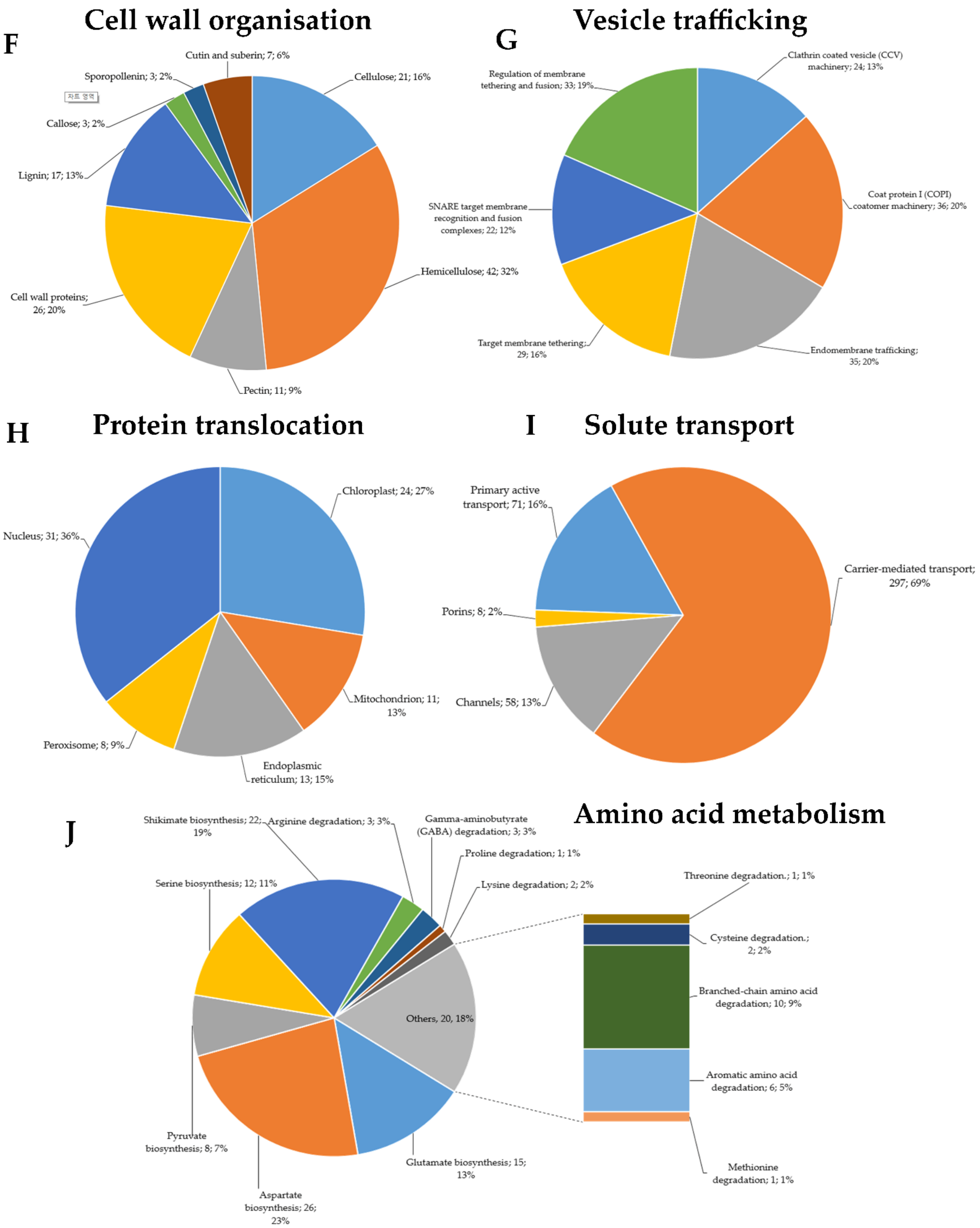
F
Cell wall organisation
Cutin and suberin; 7; 6%
Sporopollenin; 3; 2%
Cellulose; 21; 16%
Callose; 3; 2%
Lignin; 17; 13%
Hemicellulose; 42; 32%
Cell wall proteins; 26; 20%
Pectin; 11; 9%
G
Vesicle trafficking
Clathrin coated vesicle (CCV) machinery; 24; 13%
Regulation of membrane tethering and fusion; 33; 19%
Coat protein I (COPI) coatomer machinery; 36; 20%
SNARE target membrane recognition and fusion complexes; 22; 12%
Target membrane tethering; 29; 16%
Endomembrane trafficking; 35; 20%
H
Protein translocation
Chloroplast; 24; 27%
Nucleus; 31; 36%
Mitochondrion; 11; 13%
Peroxisome; 8; 9%
Endoplasmic reticulum; 13; 15%
I
Solute transport
Primary active transport; 71; 16%
Carrier-mediated transport; 297; 69%
Porins; 8; 2%
Channels; 58; 13%
J
Amino acid metabolism
Shikimate biosynthesis; 22; 19%
Arginine degradation; 3; 3%
Gamma-aminobutyrate (GABA) degradation; 3; 3%
Proline degradation; 1; 1%
Lysine degradation; 2; 2%
Serine biosynthesis; 12; 11%
Threonine degradation.; 1; 1%
Cysteine degradation.; 2; 2%
Branched-chain amino acid degradation; 10; 9%
Others, 20, 18%
Aromatic amino acid degradation; 6; 5%
Pyruvate biosynthesis; 8; 7%
Glutamate biosynthesis; 15; 13%
Methionine degradation; 1; 1%
Aspartate biosynthesis; 26; 23%

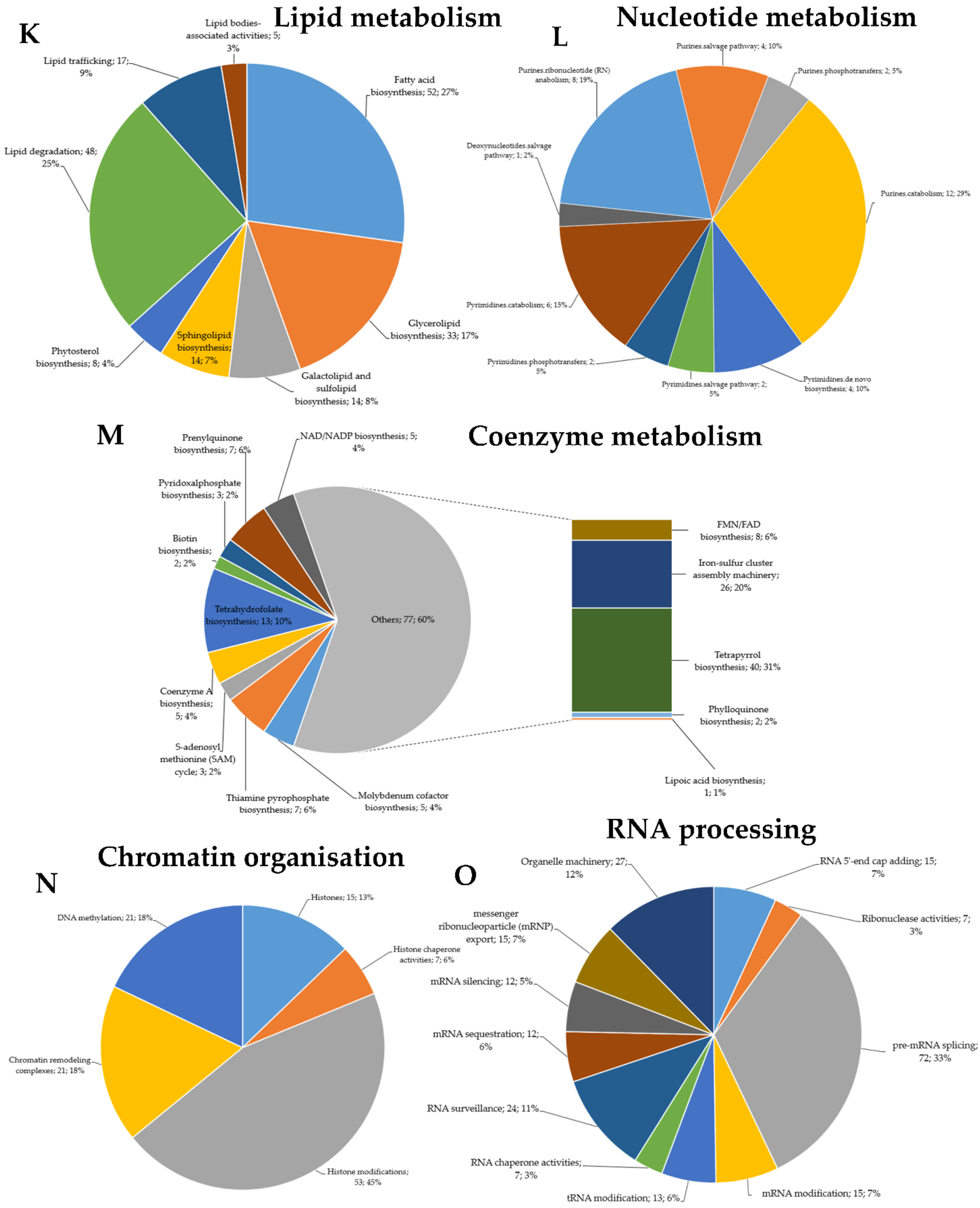

Figure S2. Regulatory and signaling pathways triggered by GRH–mediated biotic stress in rice. (**A**) Differential expressed genes (DEGs) involved in multi–process regulation (i.e. Programmed Cell Death, Circadian clock systems, SnRK1–kinase regulatory system, etc.), (**B**) protein modification (i.e. *S*–nitrosylation, *S*–glutathionylation, Acetylation, Disulfide bonding, etc.), (**C**) Cell cycle organization (i.e. DNA replication, organelle division, mitosis, etc.), (**D**) nutrient uptake, (**E**) protein homeostasis (i.e. Ubiquitin deconjugation, ER–associated protein degradation, etc.), (**F**) cell wall organization (i.e. Cutin, ligning, hemicellulose, etc.), (**G**) vesicle trafficking (regulation of membrane tethering and fusion, coat protein I coatomer machinery, etc.), (**H**) protein translocation, (**I**) solute transport (Porins, primary active transport, etc.), (**J**) amino acid metabolism, (**K**) lipid metabolism, (**L**) nucleotide metabolism, (**M**) coenzyme metabolism, (**N**) chromatin organization (i.e. DNA methylation, histone chaperon activity, etc.), and (**O**) RNA processing (i.e. mRNA sequestration, Ribonuclease activity, mRNA modification, etc.).

Table S1. Electrical penetration graph (EPG) results.

Rice Lines	No Insects	EPG Parameters (6 h)		
		Nc2–Nc6 (min)	Cumulative Nc6 (min)	Nc2–Nc5 (min)
Ilpum (Recurrent, GRH susceptible)	1	80.92	249.57	48.32
	2	87.48	229.29	0.85
	3	68.69	162.81	14.81
	4	74.00	249.65	38.53
	5	47.59	236.62	24.77
	6	34.22	297.32	26.97
	7	142.58	16.53	11.36
	8	67.34	291.63	23.65
	9	288.19	52.35	267.03
	10	359.70	0.00	48.25
Shingwang (Donor, *Grh1*)	1	358.51	0.00	14.35
	2	358.94	0.00	14.03
	3	359.19	0.00	40.44
	4	359.42	0.00	27.48
	5	359.46	0.00	57.85
	6	359.63	0.00	13.56
	7	359.68	0.00	34.49
	8	356.93	0.00	104.23
	9	357.99	0.00	357.99
	10	357.95	0.00	140.88
Near Isogenic Line (Carrying *Grh1*)	1	357.98	0.00	45.04
	2	357.08	0.00	357.08
	3	358.52	0.00	28.30
	4	330.27	0.00	16.00
	5	359.71	0.00	106.08
	6	359.68	0.00	48.35
	7	358.39	0.00	53.05
	8	358.67	0.00	358.67
	9	358.08	0.00	36.65
	10	359.18	0.00	33.18

Table S2. List of top 20 up-regulated genes (Ilpum : 10 and Near Isogenic Line: 10) in the GRH-mediated RNA-Seq leaf transcriptome in Rice.

No.	Accession Number	MSU ID	FPKM (Val_1) Control	FPKM (Val_2) GRH	Log2 (Val_2/Val_1)	Annotation
**Upregulated genes in Ilpum**						
1	Os05t0112000-05	LOC_Os05g02130	0.000099	67.0610	19.3696	Ankyrin repeat domain-containing protein 28, putative, expressed
2	Os04t0656100-03	LOC_Os04g56160	0.006576	336.1263	15.6414	Plasma membrane ATPase, putative, expressed
3	Os06t0199200-06	LOC_Os06g09890	0.000247	2.034055	13.0076	smr domain containing protein, expressed
4	Os03t0177900-02	LOC_Os03g08050	0.011524	72.97934	12.6286	Elongation factor 1-alpha/Tu, putative, expressed
5	Os11t0106400-03	LOC_Os11g01510	0.009431	28.47166	11.5598	Ubiquitin-activating enzyme E1, putative, expressed
6	Os02t0792800-02	LOC_Os02g54980	0.065882	169.8183	11.3318	Pheophorbide a oxygenase, chloroplast precursor, expressed
7	Os01t0814400-03	LOC_Os01g59900	0.029382	52.0176	10.7899	Similar to 91A protein (Fragment), expressed protein
8	Os01t0549400-04	LOC_Os01g36860	0.026683	42.0417	10.6217	DEAD-box ATP-dependent RNA helicase 40, putative, expressed
9	Os06t0232000-01	LOC_Os06g12580	0.151176	217.1718	10.4884	Pro-resilin precursor, putative, expressed
10	Os03t0300600-03	LOC_Os03g18870	0.044299	55.9723	10.3032	Heat shock protein DnaJ, putative, expressed
**Upregulated genes in Near Isogenic Line (Shingwang × Ilpum)**						
11	Os02t0519900-04	LOC_Os02g32030	0.01689	738.9366	15.4170	Elongation factor, putative, expressed
12	Os06t0199200-06	LOC_Os06g09890	0.00032	4.0628	13.6321	smr domain containing protein, expressed
13	Os02t0792800-02	LOC_Os02g54980	0.046639	324.3186	12.7636	pheophorbide a oxygenase, chloroplast precursor, expressed
14	Os12t0168900-03	LOC_Os12g07140	0.039781	248.9986	12.6118	Similar to Vacuolar ATP synthase 16 kDa proteolipid subunit (EC 3.6.3.14)

						(V- ATPase 16 kDa proteolipid subunit), expressed protein
15	Os03t0645100-03	LOC_Os03g44300	0.002996	18.2941	12.5761	Transketolase, putative, expressed, Similar to pyruvate dehydrogenase E1 component subunit beta
16	Os01t0835600-02	LOC_Os01g61890	0.009937	56.7570	12.4797	AT hook, DNA-binding, conserved site domain containing protein
17	Os04t0636900-01	LOC_Os04g54440	0.006022	33.0418	12.4218	RNA-binding region RNP-1 (RNA recognition motif) domain containing protein
18	Os06t0686400-01	LOC_Os06g47200	0.005049	22.1922	12.1018	LTPL85 - Protease inhibitor/seed storage/LTP family protein precursor, expressed
19	Os12t0559200-01	LOC_Os12g37260	1.117746	3825.3091	11.7408	Lipoxygenase 2.1(EC 1.13.11.12), chloroplast precursor, expressed
20	Os01t0837300-01	LOC_Os01g62020	0.007443	22.231520	11.544435	NAD dependent epimerase/dehydratase family domain containing protein, expressed

Table S3. List of top 20 downregulated genes (Ilpum :10 and Near Isogenic Line: 10) in the GRH-mediated RNA-Seq leaf transcriptome in Rice.

No.	Accession Number	MSU ID	FPKM (Val_1) Control	FPKM (Val_2) GRH	Log2 (Val_2/Val_1)	Annotation
**Downregulated genes in Ilpum**						
1	Os04t0269600-02	LOC_Os04g20164	80.41164	0.000021	-21.86858	Amine oxidase precursor, putative, expressed
2	Os04t0683900-02	LOC_Os04g58730	37.45429	0.000041	-19.80108	AT hook motif domain containing protein, expressed
3	Os07t0139400-03	LOC_Os07g04690	109.41285	0.008156	-13.71156	NAD(P)-binding domain containing protein. Similar to UDP-arabinose 4-epimerase 1, putative, expressed

4	Os04t0462500-04	LOC_Os04g38870	58.72669	0.025564	-11.16569	14-3-3-like protein GF14-6, putative, expressed
5	Os04t0623300-04	LOC_Os04g53190 LOC_Os04g53195	87.93532	0.038335	-11.16356	CPuORF12 - conserved peptide uORF-containing transcript, expressed; Similar to H0215F08.3 protein, expressed protein
6	Os02t0197600-03	LOC_Os02g10390	260.99316	0.132475	-10.94408	Chlorophyll a/b-binding protein type III, putative, expressed
7	Os03t0592500-02	LOC_Os03g39610	433.82007	0.260273	-10.70286	Similar to Photosystem II type II chlorophyll A-B binding protein
8	Os04t0307200-02	LOC_Os04g24180	20.14316	0.0151	-10.38153	Heat shock protein binding protein, putative, expressed; Similar to Cysteine string protein (CCCS1)
9	Os09t0526600-03	LOC_Os09g35790	34.70458	0.071433	-8.92432	HSF-type DNA-binding domain containing protein, expressed; Similar to Heat stress transcription factor B-2c
10	Os03t0856500-01	LOC_Os03g63950	864.31470	2.346966	-8.52462	Plastid-specific 30S ribosomal protein 1, chloroplast precursor, putative, expressed(Ribosomal protein 1) (PSRP-1)
**Downregulated genes in Near Isogenic Line (Shingwang × Ilpum)**						
11	Os03t0778100-03	LOC_Os03g56670	397.14478	0.017906	-14.43693	Photosystem I reaction center subunit III, chloroplast precursor
12	Os01t0839700-01	LOC_Os01g62244	18.04017	0.001703	-13.37085	Ubiquitin-conjugating enzyme, putative, expressed
13	Os02t0820700-01	LOC_Os02g57510	17.67560	0.003447	-12.32413	SNARE domain containing protein, putative, expressed
14	Os07t0141400-01	LOC_Os07g04840	1189.69775	0.469533	-11.30708	PsbP, 23 kDa polypeptide of photosystem II, putative expressed
15	Os12t0420400-02	LOC_Os12g23200	874.95789	0.346864	-11.30063	Photosystem I reaction center subunit XI, chloroplast precursor (PSI-L) (PSI subunit V)

16	Os03t0177900-02	LOC_Os03g08050	23.71970	0.025197	-9.87862	Elongation factor 1-alpha/Tu, putative expressed
17	Os01t0869000-01	LOC_Os01g64870	41.21960	0.050321	-9.67795	Protein of unknown function DUF639 family protein
18	Os08t0416000-02	LOC_Os08g32080	9.23362	0.042114	-7.77645	CPuORF27 - conserved peptide uORF-containing transcript, expressed; Similar to Homebox-leucine zipper protein HOX5
19	Os08t0430500-03	LOC_Os08g33370	13.60227	0.067684	-7.65082	14-3-3-like protein GF14-C, putative, expressed
20	Os05t0208000-02	LOC_Os05g11780	2.58967	0.014508	-7.47977	Mitochondrial 2-oxoglutarate/malate carrier protein

Table S4. Comparative transcriptome profile by regulatory or signaling pathways between Ilpum and Near Isogenic Line under GRH–infestation.

BIN ID	Description	Ilpum							Near Isogenic Line (NIL: Shingwang x Ilpum)						
		DEGs	Expression		Downregulated		Upregulated		DEGs	Expression		Downregulated		Upregulated	
		Total	Downregulated	Upregulated	Min Log2FC	Max Log2FC	Min Log2FC	Max Log2FC	Total	Downregulated	Upregulated	Min Log2FC	Max Log2FC	Min Log2FC	Max Log2FC
1	Photosynthesis	170	117	53	–10.944	–0.013	0.003	6.690	170	145	25	–14.437	–0.196	0.081	8.113
2	Cellular respiration	95	31	64	–5.748	–0.010	0.099	7.526	95	45	50	–4.194	–0.075	0.021	5.283
3	Carbohydrate Metabolism	130	67	63	–13.712	–0.038	0.235	6.927	130	60	70	–5.413	–0.003	0.017	11.544
4	Amino acid metabolism	112	55	57	–6.301	–0.079	0.068	8.584	112	40	72	–3.057	–0.038	0.019	6.445

5	Lipid metabolism	191	90	101	–3.701	–0.048	0.004	7.867	191	79	112	–6.119	–0.011	0.007	8.032
6	Nucleotide metabolism	41	22	19	–2.493	–0.019	0.138	4.394	41	19	22	–3.216	–0.059	0.031	7.827
7	Coenzyme metabolism	127	74	53	–6.105	–0.035	0.096	6.614	127	66	61	–3.685	–0.038	0.056	6.594
8	Polyamine metabolism	14	7	7	–11.164	–0.697	0.117	4.647	14	3	11	–0.357	–0.161	0.028	0.028
9	Secondary metabolism	64	17	47	–3.785	–0.016	0.035	8.584	64	17	47	–2.719	–0.171	0.032	9.105
10	Redox homeostasis	54	27	27	–4.962	–0.259	0.017	5.038	54	30	24	–6.681	–0.029	0.015	2.593
11	Phytohormone action	168	68	100	–7.283	–0.006	0.010	7.334	168	50	118	–4.875	–0.006	0.013	11.741
12	Chromatin organisation	117	71	46	–3.900	–0.026	0.066	9.601	117	83	34	–6.272	–0.044	0.000	6.674
13	Cell cycle organisation	72	30	42	–3.261	–0.295	0.044	5.113	72	43	29	–5.110	–0.007	0.052	4.982
14	DNA damage response	22	13	9	–3.429	–0.584	0.279	2.635	22	14	8	–4.107	–0.123	0.048	6.623
15	RNA biosynthesis	599	305	294	–8.924	–0.001	0.005	8.417	599	243	356	–7.776	–0.007	0.006	9.986
16	RNA processing	219	135	84	–5.967	–0.001	0.029	5.903	219	141	78	–5.391	–0.004	0.017	9.948
17	Protein biosynthesis	233	92	141	–8.525	–0.006	0.010	12.629	233	101	132	–9.879	–0.009	0.008	8.800
18	Protein modification	496	220	276	–8.277	0.000	0.006	7.779	496	212	284	–6.840	–0.007	0.016	11.073
19	Protein homeostasis	434	238	196	–5.100	–0.011	0.005	19.370	434	206	228	–13.371	–0.004	0.001	9.046
20	Cytoskeleton organisation	93	47	46	–4.132	–0.070	0.032	4.984	93	44	49	–5.568	–0.014	0.030	9.212

21	Cell wall organisation	130	43	87	–5.565	–0.032	0.005	5.643	130	35	95	–2.918	–0.037	0.025	10.049
22	Vesicle trafficking	179	91	88	–4.142	–0.031	0.013	9.893	179	77	102	–12.324	–0.003	0.029	5.730
23	Protein translocation	87	45	42	–10.382	–0.016	0.026	11.332	87	51	36	–6.128	–0.002	0.035	12.764
24	Solute transport	434	201	233	–6.042	–0.012	0.001	15.641	434	171	263	–7.480	–0.001	0.025	12.612
25	Nutrient uptake	54	21	33	–4.859	–0.213	0.156	3.828	54	17	37	–6.664	–0.078	0.005	5.903
26	External stimuli response	58	29	29	–2.828	–0.014	0.040	3.536	58	33	25	–4.865	–0.036	0.032	4.147
27	Multi-process regulation	80	37	43	–3.119	–0.098	0.005	6.793	80	40	40	–2.869	–0.091	0.028	8.937
35	Not assigned	4222	2091	2131	–21.869	–0.003	0.000	13.008	4222	1997	2225	–9.678	–0.001	0.001	15.417

Table S5. List of primer sets used for gene expression analysis by qPCR.

Accession No.	Locus	Forward Primer (5′->3′)	Reverse Primer (5′->3′)	Tm °C (F/R)	G-C Contents % (F/R)	Amplicon Size (bp)
**Ilpum Up-regulated genes (RNA-Seq)**						
Os01t0793300-01	LOC_Os01g58100	GAACCGTGGAGCTACAACCA	CCCACAGCCGATCGATGTTA	59.9/59.9	55/55	150
Os05t0427400-00	LOC_Os05g35290	TTCTGCGAGGTGATGAACGG	GTACGAGCTGCCTTCCAAGA	62/62	55/55	123
Os04t0518400-01	LOC_Os04g43800	ACCCTGTGACCAACCATGTC	GCGATCAAGAACGTCGAGGA	59.9/60.2	55/55	133
**Ilpum down-regulated genes (RNA-Seq)**						

Os05t0491100-01	LOC_Os05g41210	GAAC-GGTTTCATCTCGGCTG	GTCAGCCTCAC-GGATCATCT	59.3/59.3	55/55	100
Os06t0608700-01	LOC_Os06g40640	CGGGA-TAGTGTTCCTCTCCG	CCAGCTT-GTTCATGGCGTTC	59.3/60.1	60/55	74
Os03t0146400-01	LOC_Os03g05310	CGGCAC-GCCAAAAATCTTCA	CCATCACGGG-CATTTTCGTC	60/62	50/55	85
**Up-regulated genes in Near Isogenic Line (RNA-Seq)**						
Os01t0837300-01	LOC_Os01g62020	CACAGAGGTGGTGGTGTTGA	TAGGCTGTCTAC-GCAATGCC	59.8/60.2	55/55	124
Os04t0518400-01	LOC_Os04g43800	ACCCTGTGAC-CAACCATGTC	GCGATCAAGAAC-GTCGAGGA	59.9/60.2	55/55	133
Os12t0559200-01	LOC_Os12g37260	TTGTCG-GAGGTCGTCAATGG	CATTCTTGTGCCGCG-TAAGG	60/59.9	55/55	82
**Down-regulated genes in Near Isogenic Line (RNA-Seq)**						
Os01t0839700-01	LOC_Os01g62244	GAAA-GATCTCGCGAA-GCACC	TTGTGACCTGCCAC-TCGAAG	59.4/60.3	55/55	83
Os07t0141400-01	LOC_Os07g04840	TACGAG-GACAACTTCGACGC	CCGAACTCGGTGATGGTCTT	60.1/59.8	55/55	80
Os12t0274700-01	LOC_Os12g17600	GTAC-CTGCTCCGTTCCAAGT	CTGCCATCGTAG-TATCCGGG	59.7/59.8	55/60	99
Differentially expressed genes between Ilpum and NIL **Near Isogenic Line (RNA-Seq)**						
Os07t0529000-01	LOC_Os07g34520	TACGACAGGGTGCTCAAGAC	TCCATGAGCCCTTGAACTGC	59.4/60.32	55/55	94
Os03t0820500-01	LOC_Os03g60580	CGAAGGGTTCAAGAAGGAGC	TAGGAGGTGTGGTCCTTGAGC	60.54/60.17	55/55	99
Os10t0517500-02	LOC_Os10g37340	TCATCGCAGGTAAGGACGAA	AGCGTGTCAAGCGTGTGTAT	59.82/60.18	50/50	102

Os01t0975900-01	LOC_Os01g74450	ATCGTCATGACCTTCGGCCT	AGTGGTTGTCCCAGACTCCG	59.74/60.00	55/55	105
Os02t0649300-01	LOC_Os02g43330	ATCACCTAGACTACTTGGGCG	TGCGTTCCATTCCACCATTG	59.97/59.11	52/50	95
Os01t0160800-01	LOC_Os01g06740	TGAACGGGTGGAAGGTCTTG	CCACCTTGTTGTGCTTGGAC	59.98/59.61	55/55	93
**Housekeeping gene**						
Rice_Actin1	LOC_Os05g36290	CTAGCGGTCGAACAACTGGT	ACCGGAGGA-TAGCATGAGGA	57.5/57.5	55/55	102
